# Bioactive compounds for human and planetary health

**DOI:** 10.3389/fnut.2023.1193848

**Published:** 2023-07-17

**Authors:** Martin Kussmann, David Henrique Abe Cunha, Silvia Berciano

**Affiliations:** ^1^Kompetenzzentrum für Ernährung (KErn), Freising, Germany; ^2^Kussmann Biotech GmbH, Nordkirchen, Germany; ^3^Ideatomik Creative Industries, Botucatu, Brazil; ^4^Institute of Biosciences, São Paulo State University, Rio Claro, Brazil; ^5^Friedman School of Nutrition Science and Policy, Tufts University, Boston, MA, United States

**Keywords:** bioactive, micronutrient, phytonutrient, prebiotic, probiotic, peptide, artificial intelligence, nutrition

## Abstract

Bioactive compounds found in edible plants and foods are vital for human and planetary health, yet their significance remains underappreciated. These natural bioactives, as part of whole diets, ingredients, or supplements, can modulate multiple aspects of human health and wellness. Recent advancements in omic sciences and computational biology, combined with the development of Precision Nutrition, have contributed to the convergence of nutrition and medicine, as well as more efficient and affordable healthcare solutions that harness the power of food for prevention and therapy. Innovation in this field is crucial to feed a growing global population sustainably and healthily. This requires significant changes in our food system, spanning agriculture, production, distribution and consumption. As we are facing pressing planetary health challenges, investing in bioactive-based solutions is an opportunity to protect biodiversity and the health of our soils, waters, and the atmosphere, while also creating value for consumers, patients, communities, and stakeholders. Such research and innovation targets include alternative proteins, such as cellular agriculture and plant-derived protein; natural extracts that improve shelf-life as natural preservatives; upcycling of agricultural by-products to reduce food waste; and the development of natural alternatives to synthetic fertilizers and pesticides. Translational research and innovation in the field of natural bioactives are currently being developed at two levels, using a systems-oriented approach. First, at the biological level, the interplay between these compounds and the human host and microbiome is being elucidated through omics research, big data and artificial intelligence, to accelerate both discovery and validation. Second, at the ecosystem level, efforts are focused on producing diverse nutrient-rich, flavorful, and resilient, yet high-yield agricultural crops, and educating consumers to make informed choices that benefit both their health and the planet. Adopting a system-oriented perspective helps: unravel the intricate and dynamic relationships between bioactives, nutrition, and sustainability outcomes, harnessing the power of nature to promote human health and wellbeing; foster sustainable agriculture and protect the ecosystem. Interdisciplinary collaboration in this field is needed for a new era of research and development of practical food-based solutions for some of the most pressing challenges humanity and our planet are facing today.

## 1. Introduction

Human health maintenance, especially through nutrition, cannot be uncoupled from preserving planetary health (1). Unhealthy diets, characterized by excessive caloric and inadequate nutrient consumption, are linked to an increased risk of obesity (2), diabetes (3), cardiovascular disease (4), certain types of cancer (5), and other chronic conditions (6). The prevalence of metabolic disease in developed countries has been exacerbated by the availability of cheap, energy-dense, nutrient-poor foods, as well as the promotion of sedentary lifestyles. Despite the food industry’s responsiveness to the increasing demand for products that meet the needs of health-conscious consumers, the current food system heavily relies on commodities such as corn, wheat, palm oil, and soybeans (7). This reliance has resulted in the market dominance of high-yield primary products and highly processed foods, often high in sugar, fat, and sodium, and low in essential nutrients and beneficial phytochemicals (8). Furthermore, animal agriculture is a major driver of greenhouse gas emissions, land use, and environmental degradation (9). The commercial interests protected by the current status quo are often considered a threat to the health of people and the environment, highlighting the need for aligning our food systems with our societal priorities. In other words, there is an environmental, economic, social, and ethical imperative for healthier and more sustainable food for humans, a more responsible use of planetary resources, and a reduction in the human-driven impact on climate change and biodiversity (1).

Comprehensive investigation of human nutritional health effects at the molecular level requires the understanding of the interplay between food, the gut microbiome, and the human host (10). The advent of genomics has revolutionized our understanding of the interconnections between nature and nurture, enabling the identification of gene-diet interactions, the study of the gut microbiome, and “foodomics” approaches, including mining plant genomes to predict and discover specific functional compounds with biological activity and nutritional value (11). These omic-enabled approaches offer novel opportunities to discover genes encoding bioactive proteins and peptides, or genes involved in the synthesis of phytonutrients, and prebiotics are generally the target of genome-mining approaches for discovery and innovation in the field of bioactives. While the specific targets depend on the desired benefits of the envisaged solution, the proteins and peptides of interest are typically modified or processed to create the final product, such as purified proteins or peptides, or extracts of hydrolysate with a blend of desired proteins and peptides. These products can be used as food ingredients or additives, or as nutritional supplements (12). The targeted proteins and peptides are usually not of human origin; rather, they are found in plants or other organisms usable as sources of bioactive ingredients (13). Beyond proteins and peptides, the bioinformatic tools that enable this genome mining approach are also used to identify genes that code for enzymes involved in the synthesis of phytochemicals (e.g., prebiotic oligosaccharides (14)). Once identified, these genes can be cloned and expressed in microbial systems, such as yeast or bacteria, to produce bioactive compounds in large quantities (15).

The use and integration of additional omics, such as proteomics and metabolomics, provide further insights into bioactive compounds and their effects on health. Proteomic approaches allow for the comprehensive identification and assessment of nutrition-relevant proteins, including receptors, enzymes, and transporters, shedding light on their role in mediating the impact of diet on health. Unlike metabolite concentrations, which fluctuate rapidly, protein expression in response to a dietary intervention undergoes slower changes and exhibits more prolonged effects. As a result, the timing of sample collection becomes less critical compared to metabolomics, allowing for better alignment with the specific intervention under investigation. This advantage enables a comprehensive understanding of the long-term effects of nutritional interventions on protein-level modifications and metabolic pathways. Therefore, proteomic profiling provides insights into the intricate mechanisms through which dietary interventions influence metabolism, enabling the identification and quantification of nutrition-relevant proteins (16).

Metabolomics, the high-throughput and comprehensive analysis of metabolites in a sample (e.g., bodily fluid, organ tissue, food matter) is widely used in modern nutrition research. Metabolomic data provides a snapshot of the metabolic state at any point in time, so nutrition studies using metabolomic approaches typically include metabolomic analyses at baseline as well as after the intervention or during the follow-up period. Baseline metabolomic profiles reflect the metabolic starting point, which may be useful in understanding and predicting inter-individual differences in outcomes of interest later on, while the metabolomic data collected after (or during) the intervention provides a snapshot of the individual metabolic response (16). The human gut microbiome, which is of great interest in nutrition science, encompasses a complex ecosystem in the intestine with a profound impact on host metabolism of these natural bioactives. It is being studied at the (meta)genomic level and, more recently, also at transcriptomic, proteo−/peptidomic, and metabolomic levels (17).

Overall, the effective use of omics can provide a deeper understanding of the functional consequences of dietary changes, paving the way for more targeted and effective strategies to optimize health and well-being. While modern nutrition science has made significant strides in understanding the effects of bioactive food components on human health and performance optimization [11] it is now imperative to encompass the impact on planetary health and climate change. With the projected global population set to reach 10 billion (by 2080 according to UN estimates), the urgency for more efficient and sustainable food and healthcare systems cannot be overstated. In this context, it is essential to carefully consider the environmental, economic, and social implications associated with the sourcing, production, and utilization of food and bioactives, aiming to optimize their harvest and production while minimizing undesirable impacts.

This article provides an overview of the current state of the art in bioactive compound discovery and the wide variety of applications through which this area of research can contribute to create a healthier future. The crucial role of this nexus in driving the transition towards more efficient and sustainable practices that benefit both human and planetary health is highlighted, emphasizing the interconnectedness of nutrition science, biotechnology, and environmental conservation. Special emphasis is placed on the potential of computationally enhanced discovery of natural bioactives, including the leverage of omics, artificial intelligence (AI) and other innovative methodologies to unlock new possibilities in identifying and harnessing the potential of natural bioactive compounds.

## 2. Natural bioactives

**Figure 1:**
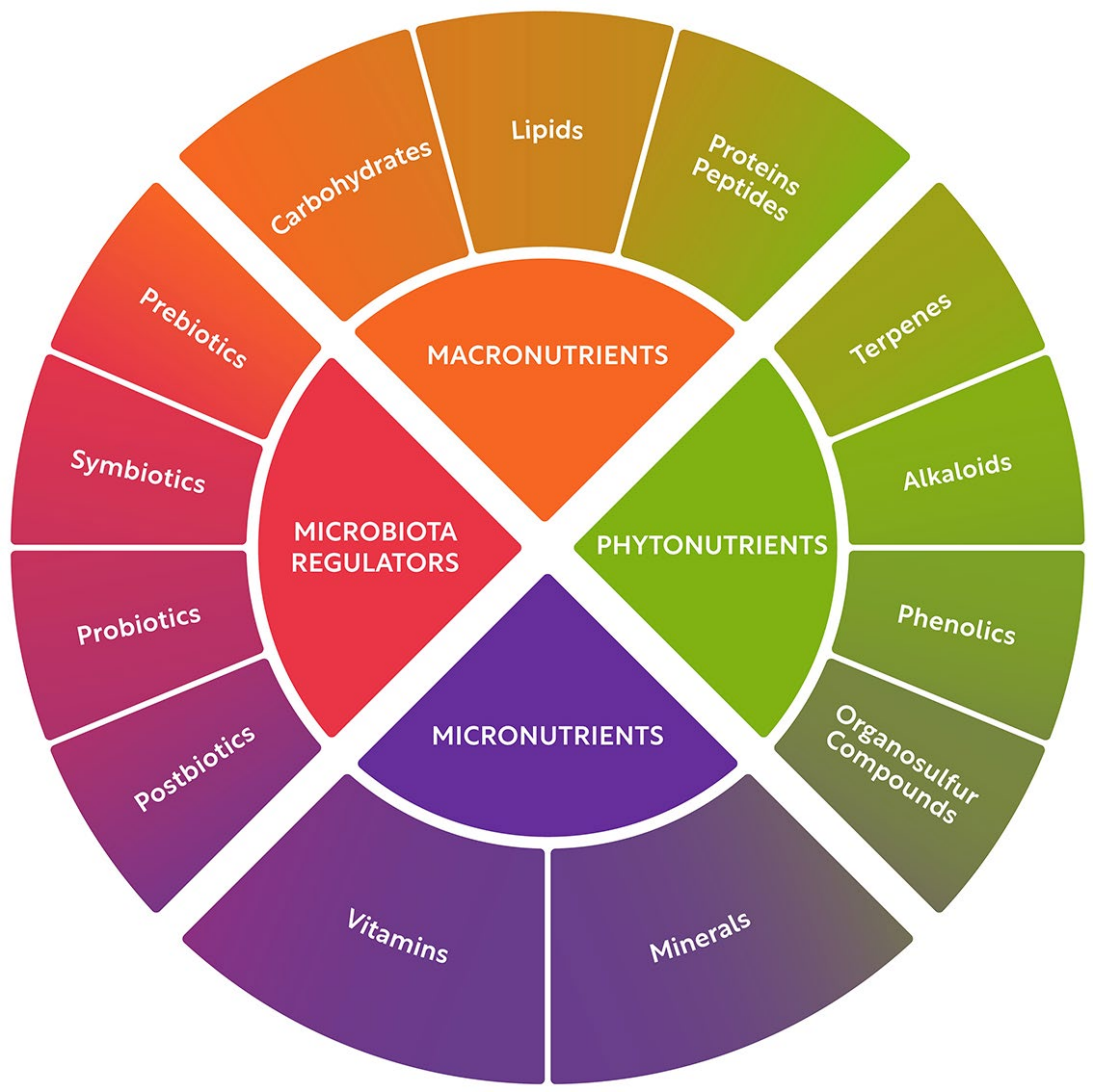
Classification of bioactive food compounds.

Nature offers a virtually unlimited source of compounds with positive effects on human health, known as natural bioactives. These compounds can be classified into four classes: macronutrients (8), micronutrients (18), phytonutrients (19), and gut microbiome regulators (20). These four bioactive classes and their major subclasses are illustrated in Figure 1. Macronutrients comprise carbohydrates, lipids, and proteins. Micronutrients include vitamins and minerals, many of which are also essential to sustain healthy body function. Phytochemicals are a heterogeneous group of minor components including terpenes, alkaloids, phenolics, and organosulfur compounds. Microbiome regulators include probiotics, prebiotics, symbiotics, and postbiotics.

Macronutrients are the major components in edible dry matter and encompass carbohydrates, lipids, and proteins. They are consumed in gram quantities per day and serve as a source of energy, building blocks, and various additional functions. Bioactive peptides chemically belong to the macronutrient “protein,” are either present *a priori* in food matter, or they are generated *in situ* during digestion of protein, or – as most important for this context – they can be released from food protein by (designed) hydrolysis.

Micronutrients are present in food in much smaller amounts and include vitamins and minerals, many of which are also essential to sustain healthy body function. These essential nutrients are consumed in milli- or micrograms quantities per day. Phytochemicals comprise a heterogeneous group of minor food components including terpenes, alkaloids, phenolics, and organosulfur compounds. Although not considered essential nutrients, they have been associated with numerous proven and potential health benefits.

Phytonutrients are natural compounds in plant foods such as vegetables, fruits, whole grain products, nuts, and legumes. Phytonutrients include phenolic compounds, alkaloids, terpenes, and other food components that are not classified as macronutrient or micronutrients, but rather as secondary metabolites in plants. These compounds can work in concert with other nutrients to exert a range of beneficial effects on human health, including antioxidant, anti-inflammatory, and neuroprotective properties (19).

Microbiome regulators include health-promoting bacterial strains known as probiotics, as well as edible food compounds that can influence the composition and activity of the gut flora, referred to as prebiotics. These prebiotics compounds include fibers and phenolics, overlapping with the macronutrient and phytonutrient categories. Additionally, optimized combinations of pre- and probiotics, known as symbiotics, as well as postbiotic products (including microbially-derived short chain fatty acids, enzymes, and vitamins) are also considered part of the microbiome regulator class.

Despite the rich natural reservoir of bioactives, both discovery and translation of natural bioactives into drugs or ingredients for pharmaceutical or nutritional applications, have faced challenges and perceptions that have hindered progress in this area (21):

- The process of discovering and developing natural bioactive-based drugs and ingredients is often perceived as slow and inefficient.
- The most easily discoverable and stronger natural bioactive-health associations are often claimed to have already been identified.
- The complex structures of natural products can pose difficulties and high costs for synthesis, particularly in the food industry where profit margins are smaller compared to the pharmaceutical sector.
- Ensuring a sustainable and standardized supply of the source material can be challenging.
- Natural product compositions can be difficult to protect as intellectual property.
- High-throughput and combinatorial chemistry are often viewed as more efficient than natural product harvesting.

However, recent insights have provided counterarguments to several challenges and limitations:

- Secondary metabolites of natural compounds have been shown to possess bioactivity.
- The structures of potentially active compounds are not limited to the imagination of scientists.
- The “Lipinski’s rule of five” (22) do not necessarily apply to natural products. These rules summarize the biophysical properties required for a drug candidate to be active after oral administration; stipulating that compounds should have a molecular weight > 500 Da, <5 hydrogen bond donors, <10 hydrogen bond acceptors, and octanol–water partition coefficient (cLogP) < 5. Lipinski himself explicitly excluded natural products from these rules due to the reasons mentioned above (22).

### 2.1. Macronutrients

Macronutrients, which include carbohydrates, lipids, and proteins, are essential for human health. The appreciation of the role of macronutrients has evolved from a more reductionistic view that sees them primarily as building blocks and energy sources, exemplified by former dogmas (e.g., “a calorie is a calorie”) and the vilification of entire macronutrient classes (lipids in the 1980s and 1990s, carbohydrates such as sugars and starches at the present time) to a more nuanced appreciation of the variety of dietary components that make up this class, as part of a more holistic understanding of diet-health relationships. This increasingly complex picture is currently focused on promoting healthy dietary patterns considering nutrient quantity and quality in the context of the whole diet and individual needs, evolving towards more personalized approaches. There are excellent examples of bioactives in each of the three macronutrient categories: ω-3 fatty acids, commonly found in fish and certain plant sources, have been extensively studied for their beneficial effects on cardiovascular health, brain function, and anti-inflammatory properties; β-glucans, i.e., fibers found in foods such as oats and mushrooms, are bioactive carbohydrates with recognized benefits for cardiovascular health. Prebiotic fibers are discussed below in the microbiome regulators section.

Bioactive food peptides belong to the protein macronutrient class, and they have unique characteristics that deserve additional discussion. Bioactive peptides can be considered the “language of nature.” All living systems use peptides to regulate and fine-tune their functions and communicate internally and externally. They have co-evolved with mankind as modulators of human physiology, typically exerting highly specific biological functions (23–25). While some bioactive peptides exist in their free form within various organisms such as humans, animals, plants, or microorganisms, the majority of known bioactive peptides are encrypted within their parent proteins. These peptides can be released through enzymatic or proteolytic processes. They can be present *a priori* in food matter, generated *in situ* during digestion of protein, or released from food protein by (designed) hydrolysis. Natural bioactive peptides derived from plants and foods are typically safe and can be used as efficacious health-beneficial active principles and ingredients. Both natural and non-natural bioactive peptides can be chemically synthesized, and play diverse roles in human health, influencing the digestive, endocrine, cardiovascular, immune, and nervous systems. They have the potential to enhance the treatment of various diseases and disorders, contributing to improved health and well-being. The growing interest in this bioactive class has incentivized the nutritional and medical scientific community, as well as the food and drug industry, to develop new functional products based on these peptides (26).

Unlike other nutrients, peptides are directly encoded in the genome, which enables their presence in natural sources and their biological functions to be predicted using computational methods. This is a unique characteristic that sets them apart from other nutrients and makes them highly amenable to *in silico* discovery (27). Peptides found in common protein hydrolysates, which are long-term consumed food sources, are generally recognized as safe (GRAS) due to their extensive safety assessments in pre-clinical and clinical studies. Consequently, they are regulated as food hydrolysates rather than drugs (28). However, this does not necessarily apply to single, or few bioactive peptides potentially enriched in food or ingredients. The challenge of using peptides as orally delivered bioactives may lie in their stability, bioavailability, and bioefficacy, rather than in their safety. Despite their potential, their health-promoting benefits remain poorly understood and utilized. This is due to the perception that most peptides have poor bioavailability, although this is not always the case [e.g., milk-derived peptides such as lactokinins (29)]. The transport of peptides from the gut lumen to the bloodstream across the gut membrane is also considered limited, although novel approaches that may enhance their digestibility and absorption are increasingly explored (30). Peptides typically have a short half-life (23), a challenge of oral peptide delivery that remains independent of the peptide format, be it a functional food product, a food ingredient, or a supplement. Additionally, the development of peptide-based drugs and ingredients has suffered from inefficient discovery and translation based on *ad hoc* research and high-throughput screening (HTS) (31).

The discovery and development of peptide-based functional food ingredients is expected to enhance high-quality protein nourishment for consumers and patients beyond a favorable amino acid supply and high digestibility, namely by delivery of additional peptide-based functionalities (32). Such functionalities range from specific health benefits to reduction or substitution of artificial food additives that are used for, e.g., preservation (33) or enhanced sensory perception. Likewise, non-natural feed additives, including antibiotics, currently still used in large-scale animal farming, may be reduced, or replaced by more sustainable and specific bioactive peptides from plant sources (34). Nutritional applications of specific peptides are currently prominent in various food product categories such as in infant formulae, sports nutrition, and dietary supplements. Peptides derived from food sources have been found to confer health benefits and provide sensory and conservation advantages in different food products. For example, fermented products (e.g., yogurt, kefir, tempeh, tofu, natto, and pickled vegetables) contain peptides that contribute to their health benefits, while peptides derived from hydrolyzed vegetable proteins enhance the savory flavors, and anti-microbial peptides produced during cheese making help protect the food from spoilage. In the future, food-derived peptides may offer solutions for reducing sugar and salt content, and aiding in the production of cultured meat proteins, and removing or replacing artificial preservatives (33).

In the past, the discovery of natural bioactives, including peptides, relied heavily on serendipitous findings. Now, the advent of HTS has significantly improved the speed and efficiency of the discovery process (35). HTS aims to capture and leverage the molecular complexity and diversity of nature, particularly in plants, through large-scale and highly automated approaches (36). Despite these advancements, translating molecular discoveries into practical solutions for consumers and patients has proven to be challenging (27).

In the field of nutrition, unlike in pharmaceutical applications, the compound purity does not necessarily correlate with its bioactivity and efficacy. Food bioactives often exert multiple subtle effects that, when considered synergistically and over the long term, can lead to significant and enduring health benefits (10, 11). Furthermore, HTS represents quite a brute-force approach to harvest, fractionation, and characterization of bioactives without much upfront guidance for possible translation and application. Such workflows typically result in a (very) high number of compounds to be tested for bioactivity and bioefficacy, with such tests and assays being lower in throughput than the upstream chemical screening (21).

Using AI and *in silico* design for peptide discovery and development can expedite the journey from concept to solution. By defining the desired benefit, AI can predict bioactive peptides that are likely to convey such benefits and can be tested *in vitro* (12). This iterative cycle of prediction and testing can generate a manageable number of potent bioactive candidates for further validation, either *in vivo* (including human studies) (37), or in a food technology context (33). Consequently, AI has the potential to enhance bioactive peptide identification, food ingredient and dietary supplement design, manufacturing processes, and clinical validation, enabling more efficient strategies for the translation of bioactive compound discoveries into effective solutions (5, 24, 29, 38).

### 2.2. Micronutrients

During the 18^th^ and 19^th^ centuries, significant progress was made in the discovery and characterization of micronutrients. In 1928, Adolf Windaus, a renowned researcher studying sterol structures, was awarded the Nobel Prize in Chemistry for his work on vitamin D and its connection to sterols. The following year, in 1929, the Nobel Prize was awarded to Frederick Hopkins and Christiaan Eijkman for their contributions to the understanding of vitamins. The term “vitamin” itself is a combination of the words “vital” and “amines,” reflecting the belief at that time that many of these essential compounds were of amine nature (39). The discoveries of vitamins often stemmed from observations of diseases that arose due to a lack or insufficient intake of specific natural food sources. For instance, scurvy was prevalent among seafarers before refrigeration was available. The disease had been known since ancient Greek and Egyptian times and is now recognized as vitamin C deficiency (40).

The field of molecular nutrition is evolving into a systems biology approach (41). This shift moves away from reductionistic notions and binary relationships between individual micronutrients and deficiency syndromes. Instead, it recognizes that nutrients act in synergy, and the overall dietary pattern and combination of nutrient levels are crucial. Additionally, the bioavailability, bioefficacy and daily requirements of micronutrients in humans are influenced by factors such as genetic makeup (42), gut microbiome (16), and exposure over time (43). This advanced understanding also encompassed the appreciation of single “parent” vitamins constituting a range of “vitamers,” that is different sub-forms or metabolites derived from the parent molecule upon intake and metabolism, with each such vitamer exhibiting a specific bioavailability and bioefficacy (44) These principles have been incorporated into a recent intervention study designed to develop better recommendations for food bioactives for populations and individuals (43). For example, more than 30 circulating vitamin forms – among other clinical, anthropometric, and food intake variables – were measured repeatedly in a 6-week multi-vitamin supplement crossover study conducted on Brazilian children (45). The results showed positive responses to the intervention for most vitamins, with changes in clinical parameters consistent with improved metabolic health. Baseline levels of metabolites, vitamins and clinical baseline measures predicted the response to the intervention. This study contributed to the development of recommendations for optimizing vitamin levels and health parameters for individuals and provided insights into micronutrient status, genomics, and individual health.

In recent years, there has been an emphasis on utilizing metabolomics and deep phenotyping approaches to better understand nutrient-health relationships at the population level. Traditional dietary assessment methods used in nutrition research rely on self-reported intake and lifestyle data, which may be subjective and prone to bias. By contrast, metabolomic profiling allows for objective evaluation of nutritional status by measuring nutrient and metabolite profiles in body fluids. Integrating self-reported dietary intake data with objective metabolomics profiles (which reflect both intake and metabolism) and phenotypic data can contribute to more accurate health prediction models and a better understanding of the impact of diet and lifestyle on individual and population health (46).

### 2.3. Phytonutrients

While plants have long been recognized for their role in human nutrition and health, the specific compounds (e.g., fibers, micronutrients, phytochemicals) responsible for their beneficial effects and the underlying mechanisms of action are still being explored. Advancements in bioanalytical techniques and systems nutrition science open new opportunities to study these compounds, combining traditional empirical knowledge with modern scientific approaches to elucidate the role of phytochemicals in human and planetary health (47).

Although phytonutrients form a scientifically well-recognized and extensively studied class of health-promoting bioactives, we may argue that the majority of them remain to be unraveled if we acknowledge the vast biosynthetic diversity present in the plant kingdom and the historically inefficient research methods. The latter, previously reliant on serendipitous findings, have been accelerated and scaled up by means of HTS (48). However, and despite leading to the development of large repositories and resources of candidate bioactives for health applications (49), HTS has not effectively bridged the gap between compound identification, assigning health benefits, and clinical translation. This may be in part due to the “bottom-up” character of traditional discovery approaches where the product is harvested, fractionated, and characterized biochemically, before the bioactivity of the isolates/fractions/compounds can be tested *in vitro* and potential benefits and applications are identified and validated.

**Figure 2:**
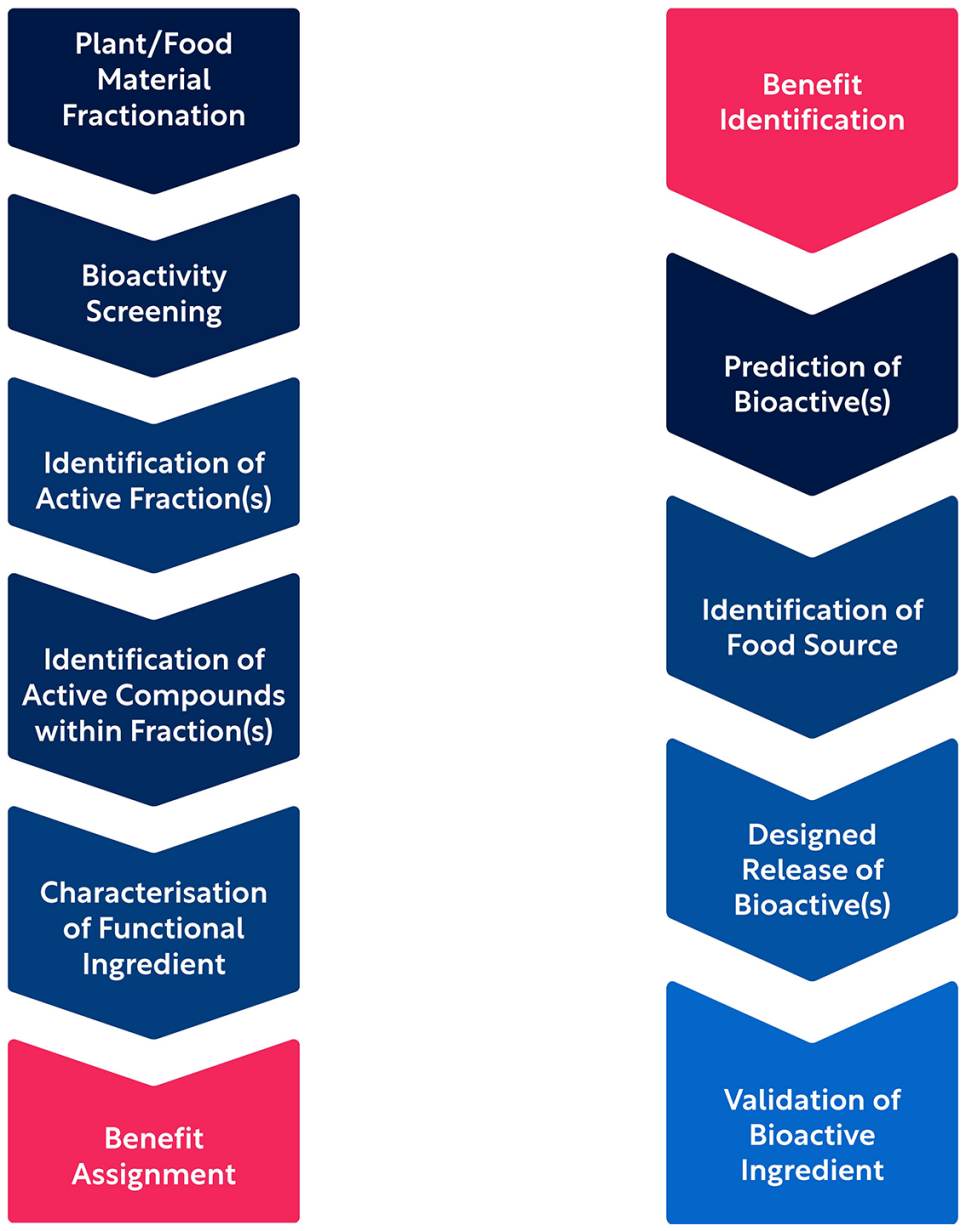
Bottom-up (left column) and top-down (right column) approaches to bioactive compound discovery and validation.

To address these challenges, novel AI approaches have been implemented in the field of phytonutrient discovery. AI enables the computational prediction of the structure, function, and sources of natural bioactives, providing a “top-down” approach to discovery (11). This design-for-benefit strategy accelerates the process of identifying and translating bioactives, significantly reducing the time from concept to prototype product (15). Such AI-driven approaches are not limited to academic research but also adopted by biotechnology companies, enabling the elucidation of the interaction between plant bioactives and human biology, prediction of bioactive plant compounds, and exploration of their impact on specific health conditions. By leveraging AI, researchers can facilitate the discovery of new phytonutrients, uncover additional benefits of existing plant-based ingredients, and identify plant sources that are rich in these beneficial bioactives (15). The process of phytonutrient discovery and validation by traditional “bottom-up” and AI-driven “top-down” approaches is depicted in Figure 2.

Polyphenols comprise plant-derived antioxidants that defend the body against ultraviolet radiation, oxidative agents, or pathogens (50). Flavanoids, lignans, stilbenes, and phenolic acids comprise the four main classes of polyphenols. Polyphenols can be found in tea, fruits, vegetables, and chocolate, and can be beneficial to human health. Dietary polyphenols help improve lipid profiles, blood pressure, insulin resistance, and systemic inflammation. Quercetin, a flavonoid, and resveratrol, a stilbene, have been associated with improved cardiovascular health. The potential therapeutic effects of dietary polyphenols might be attributed, at least partially, to bidirectional interactions with the gut microbiome (see below) (51).

Cocoa polyphenols, mainly flavanols, can modify risk factors for chronic human conditions, such as unresolved inflammation, high blood pressure, dyslipidemia, insulin resistance, and other oxidative stress-related conditions (52). Health benefits of cocoa polyphenols have been epidemiologically and experimentally documented with evidence supporting positive effects on cardiovascular, immunological, and digestive health (53). Antioxidant and anti-inflammatory effects of cocoa polyphenols were investigated in a randomized, controlled, double-blinded, crossover trial in young healthy adults who were given a single dose of high-polyphenol cocoa or maltodextrin control in separate visits, separated by a one-week wash-out period (54). Analyses of circulating metabolites, plasma antioxidant capacity and gene expression changes in peripheral blood mononuclear cells (PBMCs) were conducted both in the fasting state and 2 h post treatment. While no association was found between conjugated metabolites in plasma and antioxidant capacity, the PBMC gene expression changes suggested anti-inflammatory effects. An extension of this study aimed to compare the bioavailability of structurally related (−)-epicatechin metabolites (SREMs) after a single-dose vs. after sustained consumption of high-polyphenol cocoa. This was achieved by conducting a controlled parallel intervention trial with daily consumption of the high-polyphenol cocoa product or the maltodextrin control for 4 weeks following the crossover sequence. The individual SREM profiles and concentrations after the long-term administration and those after a single dose were found to be comparable (55).

### 2.4. The human microbiome and its regulators

Humans host various communities of bacteria, fungi, protists, archaea, and viruses. These communities are referred to as the bacteriome, mycobiome, protistome, archaeaome, and virome, respectively (56). Every mucosal surface in and on the human body is colonized and each body site within the same person has its own, distinct ecology (57). Likewise, an individual microbiome is unique and differs from the ones of other humans (58). While the human genome encodes only approximately twenty-five thousand genes, human microbiomes are estimated to encompass 2 to 20 million genes, which translates into up to 99.9% of the genetic capacity in the human body (59). Microbial communities are dynamically changing within and between each stage of life from birth to death (60) and they respond to the host’s environment. The most densely populated human organ is the gastrointestinal tract (GIT), with the colon alone harboring >70% of all microbes in the human body, dominated by bacterial phyla such as *Bacteroidetes*, *Firmicutes*, and *Actinobacteria* (57). The gut microbiome changes upon nutritional intake, both at metagenome (population census) and metabolome (functional capacity) level (61). Investigating, understanding, and leveraging the natural and induced changes in our microbiomes is primed to revolutionize our comprehension of human nutrition, biology, and health (62).

Our understanding of the gut microbiome is currently limited, with no consensus definition of what constitutes a healthy gut microbiome (20),which hinders our ability to assess the healthfulness of a gut microbiota profile based on metagenomic data or provide guidance to improve its health-promoting potential (63) in the absence of a clear picture of what target compositions may better support an individual’s health given their biological makeup, health status, diet and life stage (59). Nevertheless, pathological strains can be a sign of dysbiosis and current approaches tend to focus on reducing their presence while increasing the abundance of health-promoting bacteria such as *Bifidobacterium* and *Lactobacillus* spp. Certain probiotics have shown encouraging results in numerous preclinical studies about enhancing the prevalence of health-beneficial bacteria within the gut microbiome (64). This has raised scientific, industrial, and public interest in probiotics and prebiotics (see below) as possible agents for gut microbiome management and control in consumers and patients. Genomics, bioinformatics, and AI facilitate the establishment of mechanistic relationships within the gut microbial ecosystem, and their effects on the host’s health status (65).

Metagenomic analyses of the human gut microbiome have revealed millions of genes and genomic evidence for its benefits in human health and disease (66). Perturbation of the intestinal microbiome may result in chronic conditions such as gastrointestinal, (auto)immune, cardiovascular, and metabolic diseases (67). Via the gut-brain axis, i.e., the communication between the enteric nervous system in the gut and the central nervous systems in the brain, the intestinal microbiome has also been associated with neurological and brain disorders (68). “Association” is the correct term here though because most of the large-scale clinical microbiomics studies revealed a “coincidence” of gut microbial communities (“population census”) and human conditions, rather than any directional causality (69): in most cases it has remained elusive whether the microbiome gives rise to or is a consequence of the condition (70).

The term “gut–brain axis” (71) reflects the recognition that the enteric and autonomic nervous systems communicate, pituitary and gut hormones interact, and that these cross-talks can influence not only gut physiology but also the reward system in terms of, e.g., satiety and mood. This system can influence nerve function and, consequently, behavior in different stages of life (62). Examples for such complex and far-reaching communication are: probiotic modulation of the microbiome with impact on cholecystokinin (CCK) output, which in turn regulates vagal nerve transmissions involved in satiety regulation; and prebiotics-mediated fermentation of short-chain fatty acids (SCFAs), which triggers release of gut hormones to then in turn influence appetite regulation (64). Applications of pro- and prebiotic compounds that can induce functional (not only compositional) changes in the gut microbiome and may improve insulin sensitivity and lipid metabolism are currently pursued means for treatment of diabetes and obesity (72).

Dietary phenolic compounds can function as prebiotic modulators of the gut microbiome. Their transformation before absorption modulates their biological activity. Various studies have investigated gut microbiome-mediated transformations of polyphenols and identified the respective microorganisms. The potentially thousands of phenolic compounds in the diet are typically funneled to a much smaller number of metabolites. Selma et al. reviewed microbially generated metabolites from different phenolics and their formation pathways and the modulation of the gut microbiome population by phenolics to understand the two-way phenolic-microbiome interaction (73). Recent evidence suggests control of the gut microbiome by polyphenols including possible glycemic regulation. A good example of such polyphenol-microbiome interactions are resveratrol and curcumin, two natural antibiotics with numerous pharmacological and metabolic functions (74). Sreng et al. examined the effects of resveratrol and curcumin – alone and in association – on the control of glycemia and the microbiome. A 5-week chronic treatment of hyperglycemic mice with resveratrol and/or curcumin resulted in a differential effect on glucose tolerance and gut microbiome composition. Groups of bacteria showing a specific signature of the glycemic effect of resveratrol were identified. Metagenomic and metabolic pathway analysis plus serum metabolomics revealed that sulfur and branched-chain amino-acid metabolism correlated with the efficacy of resveratrol for glycemic control. Hence, polyphenols can specifically impact gut microbiome and its metabolic functions which may be responsible for their therapeutic role (74).

Correlations between chronological and biological age of the human host and its microbiome are also being investigated (75). Such research has revealed microbial community transformations that occur between birth and death (76); however, it remains largely unknown why specific microorganisms change abundance at certain ages. This holds true, at least in part, for the life stages from primary colonization at birth (77), changes induced by disease or antibiotics, microbial expansion at death with the successions differing by body site and microbial domain (bacteria, fungi, or viruses). Future studies of the microbiome’s relationship with age include therefore comparable, well-powered, longitudinal studies, robust statistical analyses, and improved characterization of non-bacterial microorganisms (60).

The main classes of microbiome regulators are described below.

- **Probiotics**: The specific term of “probiotic” was introduced by Vergin’s paper from 1954 entitled “Anti- und Probiotika,” in which he compared harmful effects of antibiotics and other antimicrobial agents on the intestinal microbiome with beneficial effects of selected bacteria (78). The “International Scientific Association for Probiotics and Prebiotics” (ISAPP) reinforced the FAO/WHO definition of probiotics as “live microorganisms which, when administered in adequate amounts, confer a health benefit on the host” (79). This definition captures the essential properties of a probiotic, i.e., being of microbial nature, viable, and beneficial to the host’s health, and includes a wide spectrum of microbes and applications and has been adopted by various organizations and agencies and.
- **Prebiotics**: Cheplin et al. reported in 1920 on the human intestinal microbiome being enriched with lactic bacteria after carbohydrate consumption (80). According to this original feature of prebiotics, yet considering the more recent scientific and clinical developments, the ISAPP renewed the definition of a prebiotic in 2017 (81): “a substrate that is selectively utilized by host micro-organisms conferring a health benefit.” This definition allows inclusion of non-carbohydrate substances, applications to body sites beyond the GIT, product types other than food, and use for animal health.
- **Synbiotics**: The ISAPP redefined a synbiotic in 2019 as “a mixture comprising live microorganisms and substrate(s) selectively utilized by host micro-organisms that confers a health benefit on the host” (82). This definition is more stringent than synbiotics seen simply as a mixture of pro- and prebiotics, which disregards possible cooperativity or even synergy. Perhaps one of the most meaningful actions of synbiotics have been described around human milk oligosaccharides (HMOs) and early, health-beneficial colonizers of the newborn’s gut, which is sterile at birth (83). Human milk is uniquely rich in complex carbohydrates (84). *Lactobacilli* as well as *Bifidobacteria* have been characterized as pioneering colonizers of the infant’s intestine. Combining *in vitro* microbiology for functional proof, with high-resolution tandem mass spectrometry for detailed structure elucidation, specific HMOs were identified as substrates for specific bacteria, thereby demonstrating synbiotic relationships at molecular and bacterial level (85). Such HMOs are meanwhile synthesized from (typically less complex) cow’s milk oligosaccharides (CMOs) and applied as ingredients in cow’s milk-based infant formulae to better match the nutritive value of human breast milk (86).

Independent of current and future research and treatment areas, standardized pro−/prebiotic study designs and protocols and defined test agent characteristics must be adhered to. Essentially, the quality of pro- and prebiotic research must meet the standards for either evidence-based nutrition or medicine (64). Future applications of pro- and prebiotics will extend beyond management of gastrointestinal conditions (87), because the role of the microbiome in, e.g., the peripheral and central nervous system is becoming better understood (88). Such new disease targets and treatment options may include immune (66) and mental health conditions (89).

A conceptually new perspective on discovery and design of pre-, pro- and synbiotics has recently been described, driven by the motivation to develop an immune-beneficial weaning diet for infants (90). Weaning is a period of significant physiological changes with implications for healthy early and later life: the introduction of solid foods and the changes in milk consumption are accompanied by significant gastrointestinal, immune, developmental, and microbial adaptations (77). In this study, prioritized, desired health benefits guided nutritional design upfront, which is opposed to bottom-up testing of pre−/probiotic candidate ingredients. This systems biology-based, and bioinformatics- and AI-powered methodology enabled identification of promising prebiotic combinations which in turn support growth of probiotics in the infant gut, thereby favorably influencing the development of the immune system in early life. Defining fewer infections and a better vaccine response as target health benefits for infants around weaning, the group identified *in silico* (i.e., by advanced public domain mining using computational natural language processing) infant gut microbes as potential deliverers of these benefits. They then analyzed the requirements of these bacteria for exogenous metabolites as potential prebiotics that were subsequently searched for in the natural product space. Using public domain literature mining and *in silico* reverse metabolic engineering, the team constructed probiotic-prebiotic-food associations, which guided the targeted feeding of immune health-beneficial weaning food. Competition and synergy for (prebiotic) nutrients between selected microbes was also analyzed. Finally, this information was translated into a design of an experimental complementary feed for infants enrolled in a pilot clinical trial (91), which was followed by a full trial analyzing the gut microbial changes in the infants subjected to the designed complementary feeding (92).

Biotech start-up companies and SMEs are nowadays translating this research and developing pre- and probiotic solutions for consumers and patients. The science, technology, and product portfolio of these companies ranges from pre−/probiotics prediction and discovery, via *in vitro* and *in vivo* validation to specialist formulation and production. The product formats span from dietary supplements to capsules for fecal transplantation. AI, computational network biology, and metagenomics are key enabling platforms (65).

## 3. Sustainable and efficient leverage of natural bioactive sources

It is imperative to harvest natural resources more sustainably and efficiently for sufficient macro- and micronutrient supply to the global population (93). The first objective remains to eradicate hunger by providing enough calories to the world’s still growing, yet soon predicted to be plateauing, population; the second objective is to eliminate “hidden hunger,” i.e., micronutrient undersupply (8); third, sufficient and healthier nutrition will reduce health care costs by avoidance of expensive medical and pharmaceutical interventions required for treatment of under- and malnutrition-related diseases (94), with nutrition affecting – more or less – all aspects of human health: from healthy growth via immune, gastrointestinal, metabolic, cardiovascular, endocrine health to cognitive development and performance (95). In short: prevention is more sustainable – and socially and ethically more responsible – than repair. While these overarching goals are pertinent, the means to achieve them represent “moving targets” in a rapidly changing environment: climate change is globally impacting edible crops with implications for both human well-being and the environment.

### 3.1. Sustainable alternatives to animal-derived high-quality protein

The EAT-Lancet consortium has scientifically, economically, and operationally defined both the objectives and the planetary boundaries of healthy and sustainable human nutrition (1). Humanity cannot nourish a population of 8 billion today and 10 billion predicted for 2080 based on a continued high animal protein consumption, especially in Western countries, with meat consumption growing in parallel to rising prosperity also in many other parts of the world. Beef and pork overconsumption must be reduced, for reasons of – most importantly – reducing excessive land and water use by livestock and emission of the greenhouse gas methane, and of improving human health through reduced incidence of metabolic and cardiovascular disease (96). Likewise, exploitation of protein through seafood, be it by live catch or aquafarming, is not sustainable at the current extent, with oceans being overfished and aquafarming requiring large amounts of protein feed (97).

Reducing the consumption of beef, dairy, and pork is a key sustainability priority, because meat and milk production consume more resources, e.g., land (through the cultivation of forage crops), water, fertilizer, pesticides, and energy than the production of plant-based food (98). At the same time, less greenhouse gases and nitrogen would be released into the environment. The total contribution of nutrition to global greenhouse gas emissions is approx. 25–30% (99). Various studies show that meat production from ruminants, especially cattle, will be responsible for approx. 2/3, and total animal products for around 80% of global agricultural greenhouse gas emissions in 2050 – if status and development of current eating habits do not change. Plant-based meat alternatives also offer clear advantages in terms of land and water consumption compared to conventional meat production. A diet with many plant-based foods combined with meat substitutes has an approx. 30% lower water footprint than a ketogenic, meat-heavy diet. Moreover, the practice of keeping animals in high density and large stables, which is practiced in many countries, is neither sustainable from an animal welfare perspective. Plants, insects, macroalgae (seaweed), microalgae, microbial proteins, and *in-vitro* meat are possible alternatives to animal-derived protein and are discussed hereafter.

Soybean, lentils, grains (oat, barley, quinoa, amaranth), peas, and nuts are some of the plant-based protein alternatives that do not only furnish high protein yields but also adequate protein quality, based on amino acid composition and digestibility. Soy has been traditionally and extensively grown and harvested as a plant-based protein source, especially in South America. On the Indian subcontinent, lentils are an important component of diet and serve as an affordable and sustainable protein source (100). Plant-based meat substitutes provide very different nutrient levels. Compared to meat, however, they are similarly high in protein, contain more fiber and fewer calories, less total fat, and saturated fat, but more sugar and sometimes a lot of salt. Plant-based milk drinks tend to be lower in protein, vitamins, and minerals than cow’s milk, unless fortified, yet they are usually lower in calories. Soy and oat milk for example provide health-promoting secondary plant metabolites.

According to the Food and Agriculture Organization (FAO), there are more than 1900 edible species of insects (101). Consuming insects is termed “entomophagy” and common in many cultures. In Africa, Latin America, and parts of Asia, insects are food for around 2 billion people. In 2013, the FAO launched an appeal for consuming more insects, even in Western countries where this is not or no longer common (102) Food-technological advancements are rendering such diets more palatable and attractive for consumers even if they are not culturally used to such food (103). Insects contain a lot of protein (up to 60% dry matter) and all essential amino acids. Many edible insect species are nutrient- and energy-dense and contain unsaturated fatty acids. Depending on species, age and feed, insects also provide plenty of minerals such as iron, zinc, manganese, and copper. They are therefore considered a healthy protein alternative.

Macroalgae are of great importance as food and protein supply, especially in Asia: 75–85% of the global seaweed production is used there for direct human consumption, including nori, dulse, wakame, hiziki, kelp, glasswort, or kombu algae. For food production, macroalgae are harvested from the wild or grown in aquaculture (104). The best-known microalgae are chlorella and spirulina, which are available as food supplements. Micro- and macroalgae are also rich in proteins with a high-quality amino acid profile. They contain many other nutrients such as carotenoids, vitamins, minerals, essential fatty acids, and phytochemicals (105). Macroalgae are a rich source of dietary fiber, including bioactive polysaccharides. There are numerous studies on the health effects of seaweed, which refer to anti-cancer, antiviral, antidiabetic, antihypertensive, immunomodulatory, anti-inflammatory or antioxidant properties of seaweed. Consumption of macroalgae in Asia is associated with a lower risk of cardiovascular disease, cancer, and diabetes (106). However, the bioavailability of proteins and other bioactives from algae remains unclear.

Beyond shifting cultivation, production, and consumption from animal to other sources there are also emerging technological solutions to a more sustainable food-, and especially protein supply. One of the emerging areas in nutrition innovation is the production of meat in the laboratory, known as “cellular agriculture” (107). In contrast to plant-based substitute products, meat grown *in vitro* resembles real meat tissue. It consists primarily of muscle and adipose tissue cells, while natural meat also contains other types of cells. Currently, *in vitro* meat is mainly obtained from cell cultures of beef, pork, or chicken, but there are also research efforts to produce other varieties such as fish meat in the bioreactor. There are also attempts to produce eggs, seafood, caviar, or insect meat in a test tube using carbon dioxide (108). However, scalability and sustainability remain to be proven in view of the biotechnological effort behind such production (109). *In vitro* meat is very similar to conventional meat in terms of nutritional quality (protein and fat content) and – therefore – associated health benefits and risks, because it is produced in cell culture based on animal tissue. *In vitro* meat can be enriched with those vitamins or ω-3 fatty acids that are not contained in the original product.

Precision fermentation is a rapidly growing field that involves the use of advanced biotechnological approaches to produce nutritious and health-beneficial molecules in a controlled and precise manner. This technique relies on the principles of synthetic biology and metabolic engineering to design and optimize microbial strains that can efficiently produce desired compounds. The process typically involves the genetic modification of microorganisms, such as yeast or bacteria, to introduce or enhance specific metabolic pathways that lead to the production of target molecules. Precision fermentation has several advantages over traditional methods of chemical synthesis, including greater efficiency, reduced waste, and lower environmental impact. This approach is particularly well-suited to producing complex molecules which are difficult or impossible to obtain using conventional methods. Microorganisms are used as factories in bioreactors to produce high-quality proteins, fatty acids, phytochemicals, and flavorings, or upcycle food waste into higher-value products, offering many opportunities for human and planetary health innovation. Through strain selection, screening, and engineering, coupled with relevant risk assessments, the vast biodiversity of microorganisms can be explored to create novel and safe fermented products that can benefit human health and the environment (83).

### 3.2. Micronutrients: Leveraging nutrient blends and their bioavailability

A well-balanced diet that contains diverse grains, vegetables, legumes, and fruits as well as high-quality protein-rich foods delivers sufficient micronutrients to consumers. However, large parts of the global population do not have access to such a diet. Eight hundred million people worldwide suffer from insufficient calories, and another at least 2 billion people are affected by “hidden hunger,” i.e., undernourishment in terms of micronutrients. This is still occurring for several reasons:

- Food production: cultivation of sufficient and diverse grains, legumes, vegetables, and fruits may domestically not be possible and import may be too expensive.
- Food retail: many consumers may not have physical and/or financial access to a commercially offered healthy food product selection (110).
- Climate change drives established crops into difficult growth conditions due elevated temperatures, irregular rainfall, and salty soils and groundwater because of rising sea levels flooding low agricultural land (111).
- Hence, adequate micronutrient supply cannot always be achieved through provision of balanced whole diets. Therefore, micronutrient supplements can be an – at least intermediate – affordable and logistically feasible option for compensation of what available diets may lack in terms of micronutrients (112).

An intensive area of research is increasingly analyzing many factors in agri-food systems that influence the preservation of nutrient quality which is key for nutrient security of world populations. These factors include seed varieties, seasonal and local growing conditions, transportation, food processing and storage, as well as local food customs. Modeling these systems requires data from different food system sectors including agricultural, environmental, economic, and social determinants, but also the participation of basic nutrition and biomedical science. Improving the agri-food system through advances in pre- and post-harvest processing methods, biofortification, or fortifying processed foods will help tailor nutrient requirements at both population and individual level. This challenge to maintain and improve nutrient quality is amplified by the requirement to produce food both locally and globally in a sustainable and consumer-acceptable manner (8).

Integrated “systems nutrition” research facilitates the understanding of: (i) the nutrient composition of foods; (ii) how nutrients can be preserved to deliver (fresh or processed) safe, nutritious, and affordable foods and (iii) how to optimize nutrient intake for sustaining health (113, 114). Nutrient quality is impacted by crop genetics, agricultural environment, and practices, as well as all processes from seed to fork. The loss of genetic diversity in agriculture constrains nutritional diversity and increases the plants’ vulnerability to climate change and new pests and diseases. Phenotypically flexible plants can better cope with changing environments emerging with the warming planetary atmosphere (8).

Nutrient composition is also heavily influenced by transport and processing of fresh foods. Those nutrient-affecting post-harvest activities include handling, storage, processing, transportation, and packaging. Losses are typically only captured as weight which does not fully account for specific nutrient losses. While grains, tubers, fruits, and vegetables have specific requirements for preserving their nutritional quality post-harvest, they share the sensitivities to water scarcity, physiological deterioration, mechanical damage, diseases, and pests (FAO, 1989).

Delivering the right nutrients to populations and individuals is challenging given the inconsistencies in ensuring nutritional quality across the nutrient chain. Micronutrient genomics needs to come into the equation when it comes to providing the right micronutrient blends to the right populations (115). Micronutrient bioavailability does not only depend on the nutrient content of the food source but also on genetic ancestry of the consuming individuals and populations, as nutrient-relevant enzymes and transporters, for example, underlie genetic variability, between populations and inter-individually (115). The key focus is to identify and address prevalent micronutrient deficiencies among major populations and specific groups. This entails considering crucial aspects such as available diets and the individual’s capacity to effectively acquire and metabolize essential nutrients, ultimately promoting overall well-being.

### 3.3. Phytonutrients: computational exploration of plant metabolism for biosynthetic capacity and understanding environmental impact

While phytonutrients have been extensively studied for their health effects and applicability within diets or as supplements, we are still far from efficiently, comprehensively, and sustainably leveraging the richness of these plant bioactives. To accelerate discovery and translation of such phytonutrient solutions to human, especially nutritionally actionable, conditions, we need to further develop and better deploy computational human biology and *in silico* plant metabolism, both enhanced by AI, to find the right plant molecules and connect them to matching human health applications (15). This implies a top-down approach of benefit definition and design upfront, rather than traditional bottom-up harvest of natural bioactives and subsequent high-throughput screening and testing (12).

A further challenge to be overcome is securing and standardizing supply of the raw plant material, the phytonutrient extracts, and final blends of bioactives (116). This requires stable supplier contracts, secured logistics, and robust extraction, purification, and formulation processes (117). Also, the plant source should be genetically characterized and seasonal and regional variability of the plant itself and its phytonutrient spectrum should be controlled (116).

However, even if this chain of interconnected and interdependent steps is established in a robust workflow, registration and regulatory approval may still pose challenges (118). This applies to the objective of making science- and evidence-based hard claims for functionality and efficacy of the phytonutrient blend. Regulatory authorities are more in favor of approving single or few active principles rather than complex blends. Such molecular blends are difficult to reproducibly generate and formulate, and it is challenging to unequivocally demonstrate the claimed biological functionality and health benefit as specifically linked to the particular mixture of bioactive principles (119).

Many studies have examined the effects of climate change on crop yields, yet few studies have examined effects on crop quality, such as, e.g., concentrations of phytonutrients and their secondary plant metabolites. This shortcoming was addressed in a recent review focusing on *Camelia sinensis* as a model system for investigating environmental effects of climate change on crop quality (120). The tea plant was chosen as study system due to its global revelance as a crop produced in over 50 countries on 5 continents, and because this plant that is cultivated for its quality, directly related to secondary plant metabolite profiles including catechin, caffeine, volatile secondary metabolites, plus carbohydrates and amino acids which contribute to nutritional and sensorial quality of the beverage. Health claims on tea encompass antioxidant, anti-inflammatory, anti-cancer, anti-microbial, anti-atherosclerotic, and neuro- and cardio-protective bioactivities. Besides its cultural and health relevance, tea is a good model system to examine climate change effects because of its woody perennial nature which renders tea susceptible to detrimental effects of climate change. The review found that seasonality, water, light supply, temperature, herbivory and microbes, and soil factors – all influenced by climate change – can result in up to 50% increased or decreased content of secondary plant metabolites (120). The environmental factors with the most consistent evidence in this systematic review were seasonality and water stress.

### 3.4. Unleashing the microbiome potential: from sequencing to intervention

Microbiome research and its translation into solutions for human health is advancing from large-scale sequencing and association studies to a deeper functional understanding of this prokaryotic ecosystem within the eukaryotic host and of the mutual interactions (121). This opens possibilities for intervention and treatment options for consumers and patients. These are currently being pursued through the development of pre−/pro−/synbiotics-based dietary supplements (64) and microbiome formulations for fecal transplantation (122).

However, in view of the complexity, plasticity (123), and responsiveness of the human gut microbiome, especially to diet (124), and from a sustainability perspective, pursuing a “seeding through feeding” approach may be more affordable and scalable (90): such strategy interprets – or even designs – food to seed and grow health-beneficial gut bacteria and, eventually, build a healthy, resilient microbiome based on high genetic diversity. This said, the definition of a “healthy microbiome” depends on genetic ancestry, personal lifestyle, and individual disease susceptibility. Providing healthy food not only for the human host but also for the gut microbiome appears as a pertinent concept of a more holistic and sustainable approach to nutritional health, which can contribute to the reduction of chronic, especially diet-related and -induced, disease burden with a focus on gastrointestinal, metabolic, and immune disorders.

## 4. Conclusion

The treasure of natural bioactives must be more efficiently and sustainably lifted to the benefit of human and planetary health. Natural bioactives can make a big and sustainable difference in improving human and animal health and making the food system healthier and more sustainable. Both contribute to a more efficient and affordable health care system and a more careful use of terrestrial and oceanic food resources.

Systems nutrition, computational biology, and artificial intelligence are the key enabling scientific disciplines of this transformation because they allow for an efficient, targeted “design-for-benefit” approach to complement or even replace the conventional bottom-up “find-test-and-see” strategy. This is based on the systems-level understanding of host and microbiome metabolism and on the prediction of functionality and source of bioactives. Micronutrients, phytonutrients, bioactive peptides, and pre−/probiotics should be included in this enhanced discovery, validation, translation, and validation of natural bioactives.

Taken together, we consider such translational and innovative approach to the leverage of natural bioactives as an integral part of, and key contribution to, feeding the world population more healthily and sustainably, now and for generations to come. Our described challenges and efforts in the science of bioactives must be complemented by a more sustainable, yet still highly productive agriculture, plant-forward diets that support human health within planetary boundaries, better distribution by improved logistics, and a better education of the public about nutrition and health.

## Author contributions

MK conceived the idea and concept for this article and drafted the manuscript. All authors contributed to the article and approved the submitted version.

## Conflict of interest

Author MK is CEO and Founder of Kussmann Biotech GmbH. Author DHAC is CEO and Founder of Ideatomik Creative Industries.

The remaining author declares that the research was conducted in the absence of any commercial or financial relationships that could be construed as a potential conflict of interest.

## Publisher’s note

All claims expressed in this article are solely those of the authors and do not necessarily represent those of their affiliated organizations, or those of the publisher, the editors and the reviewers. Any product that may be evaluated in this article, or claim that may be made by its manufacturer, is not guaranteed or endorsed by the publisher.
